# Caprines expressing genes of pharmaceutical applications

**DOI:** 10.1186/1753-6561-8-S4-O32

**Published:** 2014-10-01

**Authors:** Harry Meade

**Affiliations:** 1 rEVO Biologics, 175 Crossing Blvd, Framingham, MA 01702, USA

## 

The mammary gland has unparalleled capacity to produce proteins. We have harnessed this potential to produce g/L levels of recombinant proteins in milk. Using the beta casein promoter to drive expression of target proteins, kg quantities can be obtained from each goat during a normal lactation. For the past 20 years, we have produced recombinant versions of human serum proteins, (i.e. Antithrombin, Alpha-_1 _Antitrypsin, Alpha Fetoprotein, Human Serum Albumin, FactorVII).

The technology has also allowed the production of high levels of monoclonal antibodies (Mabs). These included contracts for some innovator companies to produce their antibody candidates. However, to move into the follow-on biologics arena, we have generated lines to produce our own versions of adalimumab, trastuzumab, cetuximab, etanercept and a Mab to CD20. Lactating goat lines are currently producing levels that range from 2g/L to the trastuzumab line at 40g/L.

Taking advantage of the unique glycosylation properties of the mammary gland, we have found that some of these antibodies have increased effector functions. In addition, the centuximab produced does not contain the alpha-_1,3 _gal present on the commercial product.

This capacity for production coupled with the reduced capital requirement and freedom from expression patents enable a new generation of biologicals

